# Induction of ischaemic cardiomyopathy in mice without off‐target effects

**DOI:** 10.1113/EP093310

**Published:** 2025-10-19

**Authors:** Mark T. Waddingham, James T. Pearson

**Affiliations:** ^1^ Department of Cardiac Physiology National Cerebral and Cardiovascular Center Research Institute Suita‐shi Osaka Japan

**Keywords:** apoptosis, atherosclerosis, inflammation, ischaemic heart failure, necrosis

1

Ischaemic cardiomyopathy is defined as the onset of a progressive decline in left ventricular (LV) contractile function resulting from coronary arterial disease that leads to LV chamber dilatation and diminished ability to pump blood. The rupture of coronary stenoses can now largely be avoided through percutaneous coronary intervention and intravascular stent deployment with access to cardiovascular intervention specialists. However, over the last decade or so the burden of atherosclerotic plaques in the large epicardial arteries has been demonstrated to be more likely to contribute to sudden cardiac death through plaque erosion rather than rupture, leading to the gradual release of obstructive and thrombotic materials that cause coronary microembolization and myocardial infarction. In recent years, there has been renewed interest in understanding how plaque debris that is eroded through haemodynamic events or coronary interventions leads not only to microvascular obstructions and coronary microvascular dysfunction, but also to ongoing necrosis and apoptosis with patchy microinfarct formation (Kleinbongard & Heusch, 2022). In addition to physical microvessel obstruction per se by plaque debris, platelet aggregates and fibrin, there is a central role for the release of soluble factors in driving more widespread endothelial dysfunction and vasoconstriction and the initiation of a sustained pro‐inflammatory and pro‐oxidative cascade that detrimentally impacts neighbouring cardiomyocytes (Kleinbongard & Heusch, 2022). To understand these pathological changes associated with coronary microembolization, both small and large animal models have been developed and tested many times with intracoronary (large animal models mostly) or intraventricular infusion of inert particles and microspheres, which vary considerably between studies in their ability to evoke prothrombotic, pro‐inflammatory and vasoconstrictor activity.

In this issue of *Experimental Physiology*, Pei et al. (2025) describe the development of an improved murine model of ischaemic cardiomyopathy induced by coronary microembolization through intraventricular microinjection guided by palpation of the chest to localise the heart apex. The authors successfully delivered a slow injection of insoluble corn oil in saline solution (doses between 0 and 60 µL oil in 80 µL injection volume) directly into the LV chamber in anaesthetized mice, then demonstrated a rapid onset of constriction in coronary small arteries–arterioles and obstruction of more distal microvessels through open‐chest photoacoustic microscopy of the coronary vasculature, which is based on laser activation of haemoglobin and ultrasound detection of the generated acoustic signal (Pei et al., 2025). Importantly, these authors demonstrated that the dose‐dependent reaction to the slow infusion of oil solution, which is assumed to enter the coronary arteries directly off the aortic root during blood ejection, resulted in significant suppression of LV ejection fraction and fractional shortening and LV chamber dilatation 1 day after administration. Consistent with the findings of other studies in various models, this early phase cardiac dysfunction after the ischaemic episode did not appear to involve induction of apoptosis but rather necrosis (Kleinbongard & Heusch, 2022).

Follow‐up of a cohort of mice 1 month after oil injection revealed the most progressive decline at the high (60 µL) oil dose in LV contractile function, an increase in patchy fibrosis and myocardial immune cell infiltration associated with elevated cardiomyocyte apoptosis; typical characteristics of ischaemic cardiomyopathy and progressive heart failure attributable to secondary loss of cardiomyocytes following ischaemic insults, long after microbstructions are resolved. Interestingly, LV dilatation did not appear to increase from the 1 day time point, whereas there was sustained, pronounced vasoconstriction of second‐ and third‐order coronary arterial vessels, suggesting ongoing coronary endothelial dysfunction and exaggerated vasoconstrictor activity in the mice at 1 month after oil injection. Whether this decline coronary blood flow distribution involves diminished nitric oxide signalling, as described in a spontaneous diet‐induced atherothrombotic murine model (Pearson et al., 2017), remains to be determined.

Pei et al. (2025) validated their findings first with a transcriptome comparison with an existing database for human ischaemic heart failure and second, by evaluating standard therapies in this murine model. Not surprisingly, the authors demonstrated by Gene Ontology enrichment analysis that the myocardium of this murine model showed upregulation of genes related to inflammation, collagen and connective tissue formation, leucocyte chemotaxis and vascular tone, whereas genes related to cardiomyocyte structure and contractility were differentially downregulated.

The future potential of using this oil injection approach in mice to understand the pathological progression of ischaemic cardiomyopathy to heart failure and potential therapeutic interventions can be ascertained by several notable findings from the study. Pei et al. (2025) demonstrated that two standard therapies, sacubitril–valsartan therapy (neprilysin inhibitor combined with angiotensin receptor blockade) and nitroglycerin therapy from the time of oil injection had distinct effects on ischaemic injury, by reducing the adverse dilatation of the LV (presumably, by reducing afterload) in the former treated mice and improved contractile function at 1 month after oil injection in both treatment groups. Corn oil injection did not appear to impair perfusion of other systemic organs or the brain in these mice, which contrasts with the only study in which microspheres were injected intraventricularly in mice; neither microspheres nor corn oil has prothrombotic, pro‐inflammatory or vasoconstrictor activity. Nonetheless, corn oil is amenable as a vehicle for delivery of antagonists/agonists to enhance or ameliorate these characteristics of ischaemic cardiomyopathy development.

## AUTHOR CONTRIBUTIONS

Both authors have read and approved the final version of this manuscript and agree to be accountable for all aspects of the work that questions related to the accuracy or integrity of any part of the work are appropriately investigated and resolved. All persons designated as authors qualify for authorship, and all those who qualify for authorship are listed.

## CONFLICT OF INTEREST

None declared.

## FUNDING INFORMATION

None.
